# The prediction of live weight of hair goats through penalized regression methods: LASSO and adaptive LASSO

**DOI:** 10.5194/aab-61-451-2018

**Published:** 2018-11-19

**Authors:** Suna Akkol

**Affiliations:** Biometry and Genetic Unit, Department of Animal Science, Faculty of Agriculture, Van Yuzuncu Yil University, Van, Turkey

## Abstract

The least absolute selection and shrinkage operator (LASSO) and adaptive LASSO methods have become a popular model in the last decade, especially for data with a multicollinearity problem. This study was conducted to estimate the live weight (LW) of Hair goats from biometric measurements and to select variables in order to reduce the model complexity by using penalized regression methods: LASSO and adaptive LASSO for γ=0.5 and γ=1. The data were obtained from 132 adult goats in Honaz district of Denizli province. Age, gender, forehead width, ear length, head length, chest width, rump height, withers height, back height, chest depth, chest girth, and body length were used as explanatory variables. The adjusted coefficient of determination ($R_{\mathrm{adj}}^{2}$), root mean square error (RMSE), Akaike's information criterion (AIC), Schwarz Bayesian criterion (SBC), and average square error (ASE) were used in order to compare the effectiveness of the methods. It was concluded that adaptive LASSO (γ=1) estimated the LW with the highest accuracy for both male ($R_{\mathrm{adj}}^{2}=0.9048$; RMSE = 3.6250; AIC = 79.2974; SBC = 65.2633; ASE = 7.8843) and female ($R_{\mathrm{adj}}^{2}=0.7668$; RMSE = 4.4069; AIC = 392.5405; SBC = 308.9888; ASE = 18.2193) Hair goats when all the criteria were considered.

## 1 Introduction

Native goat breeds play important socio-economic roles in the livelihood strategies of poorer farmers, especially those in rural and hard-to-reach areas of the world. Turkey has one of the largest goat populations in the world and has one of the highest breeding rates. The total number of goats in the country is about 10.3 million and the dominant goat breed is the “Common”, or “Hair”, goat, which constitutes approximately 92 % of the total goat population in the country (TUIK, 2017). Goats have been kept for milk, meat, skin, and hair for several centuries in Anatolia (Gokdal, 2013).

Studies to define adult live weights and body measurements are of great importance for the characterization of farm animal breeds. The prediction of body weight (BW) and the determination of its relationships with other biometric measurements generates considerable knowledge for breeding research relating to meat production per animal (Iqbal et al., 2013; Yılmaz et al., 2013; Khan et al., 2014). Multiple linear regression (MLR), based on ordinary least squares (OLS), is a traditional, simple method that has been used by researchers in order to predict the complex relationship between live weight and some body measurements in goat, sheep, cattle, fish, etc. (Francis et al., 2002; Pesmen and Yardimci, 2008; Yılmaz et al., 2013). However, when a multicollinearity problem exists among explanatory variables, the OLS method produces poor predictions (Montgomery et al., 2001; Yakubu, 2010; Dormann et al., 2013; Khan et al., 2014). The multicollinearity problem implies that the standard errors of regression coefficients are higher than expected, and thus it is difficult to find out the accuracy and robustness of the prediction models (Weisberg, 2005; Yakubu, 2009, 2010; Sangun et al., 2009).

Penalized methods based on minimizing the residual sum of squares are an alternative to OLS method for data with multicollinearity problems. Ridge regression is one of them; it overcomes the multicollinearity problem by using $l_{2}$-norm in order to shrink the regression coefficients (Hoerl and Kennard, 1970; Marquardt and Snee, 1975; Dormann et al., 2013). Ridge regression has been previously used by some researchers working on the prediction of live weight (Malau-Aduli et al., 2004; Yakubu, 2009; Topal et al., 2010). It works by keeping all the explanatory variables in the model; however, it cannot perform variable selection (Zou and Hastie, 2005). However, variable selection is as important as prediction in a model with a large number of explanatory variables. The other penalized method used in the current study is the least absolute shrinkage and selection operator (LASSO) proposed by Tibshirani (1996). LASSO uses $l_{1}$-norm and executes both automatic variable selection and continuous shrinkage simultaneously (Zou and Hastie, 2005; Wang et al., 2011). These properties make LASSO a popular variable selection method (Wang et al., 2011; Ogutu et al., 2012; Akkol et al., 2018). However, LASSO has some important limitations in practice (Zou and Hastie, 2005). One of them is that LASSO selects only one or a few variables and shrinks the rest to 0 if the model includes a number of correlated explanatory variables (Zou and Hastie, 2005; Wang et al., 2011). This might be an undesirable feature in many studies. Fan and Li (2001) showed that LASSO does not produce unbiased estimates for large coefficients and that LASSO does not possess oracle properties. Zou (2006) introduced the adaptive LASSO (ALASSO) estimators to remedy the problem, by adding data-defined weights to the original LASSO version. He showed that ALASSO can have oracle properties if the weights are dependent on the data and are wisely chosen. In his study, Zou used LASSO and ALASSO for γ=0.5, γ=1, and γ=2 and revealed that ALASSO is closer to the true model than LASSO and also that ALASSO for γ=1 is closer to the true model than the one for γ=0.5.

The aim of this study was to estimate the LW of Hair goats from biometric measurements for the purpose of selection for genetic improvement and breeding program in the field to select variables in order to reduce the model complexity and to determine the best model to explain the change in LW by performing ALASSO. Therefore, multiple linear regression was performed to determine a potential multicollinearity problem; then the Ridge, LASSO, and ALASSO methods for γ=0.5 and for γ=1 were compared to each other in order to obtain the best fit model.

## 2 Material and methods

### 2.1 Material

The data of the study comprised measurements from a total of 132 Hair goats from the Honaz district of Denizli province in Turkey. The data included age, gender, live weight, and 10 biometric measures of goats: forehead width (FW), ear length (EL), head length (HL), chest width (CW), rump height (RH), withers height (WH), back height (BH), chest depth (CD), chest girth (CG), and body length (BL) were recorded in the breeding season. Live weights of the goats were determined with a digital scale. CW, RH, WH, BH, CD, and BL were measured with a measuring stick, and FW, EL, HL, and CG were measured with a measuring tape.

### 2.2 Methods

The basic multiple linear regression model used to predict the live weight with the LASSO and ALASSO model:

1 $$Y=\mu 1_{n}+X\beta +e,$$

where $Y=(y_{1},y_{2}$, … $y_{n}{)}^{T}$ is a vector of observed dependent variables, $1_{n}$ is a column vector of n variables (i=1, 2, 3 …, n), μ is the intercept, X is an nxp matrix of explanatory variables, β is the vector of regression coefficients, and e is the vector of the residuals with a mean of zero and a variance $I\sigma_{e}^{2}$. It was assumed that observed independent variables have been mean-centered in regularized linear regression.

#### 2.2.1 LASSO regression

In the OLS method, β coefficients are estimated by minimizing the sum of residuals squares (RSSs). This is expressed as an optimization problem by the following equation

2 $$\hat{\beta }=\mathrm{arg}\underset{\beta }{min}|Y-X\beta {|}^{2}.$$

The following equation in the Lagrangian form is used to calculate the regression coefficients with LASSO.

3 $$\hat{\beta }(\mathrm{lasso})=\mathrm{arg}\underset{\beta }{min}|Y-X\beta {|}^{2}+\lambda |\beta {|}_{1},$$

where $|Y-X\beta {|}^{2}=\sum_{i=1}^{n}(y_{i}-x_{i}^{T}\beta {)}^{2}$ is the loss function, $\left|\beta {|}_{1}=\sum_{j=1}^{p}|\beta_{j}\right|$ is the $l_{1}$-norm penalty on β, and λ≥0 is a tuning (penalty or shrinkage) parameter which regulates strength of penalty and is important for the success of LASSO. For the LASSO estimate Eq. (3) is rewritten without an intercept (Hastie et al., 2009):

4 $$\hat{\beta }(\mathrm{lasso})=\mathrm{arg}\underset{\beta }{min}\left\{\frac{1}{2}\sum_{i=1}^{n}{\left(y_{i}-\sum_{j=1}^{p}x_{ij}\beta_{j}\right)}^{2}+\lambda \sum_{j=1}^{p}\left|\beta_{j}\right|\right\}.$$

The penalty function called $\ell_{1}$ is important for the success of LASSO.

#### 2.2.2 Adaptive LASSO regression

ALASSO modifies the original LASSO penalty by adding weights for each parameter to the penalty term. These weights are data-defined weights, ${\hat{\omega }}_{j}$, and they control the shrinking of the zero coefficients more than the non-zero coefficients. The ALASSO estimates $\hat{\beta }$(alasso) are given by

5 $$\hat{\beta }(\mathrm{alasso})=\mathrm{arg}\underset{\beta }{min}{∥y_{i}-\sum_{j=1}^{p}x_{ij}\beta_{j}∥}^{2}+\lambda \sum_{j=1}^{p}{\hat{\omega }}_{j}\left|\beta_{j}\right|$$

where ${\hat{\omega }}_{j}=1/|{\hat{\beta }}_{j}^{\mathrm{ini}}{|}^{\gamma }$ is a known weights vector, γ is a positive constant > 0, and ${\hat{\beta }}_{j}^{\mathrm{ini}}$ is the initial consistent estimator of β obtained from ordinary least square or ridge regression if there is a multicollinearity problem (Zou, 2006; Ogutu et al., 2012). When the parameter estimates produced by ALASSO are defined by $\hat{\beta }(\lambda_{n})$, then

6 $$\hat{\beta }\left(\lambda_{n}\right)=\mathrm{arg}min\left\{{∥Y-\sum_{j=1}^{p}X_{j}\beta_{j}∥}^{2}+\sum_{j=1}^{p}\lambda_{n}{\hat{\omega }}_{j}\left|\beta_{j}\right|\right\}.$$

It was proved that ALASSO has the oracle property when $\lambda_{n}\to \infty$ and $\lambda_{n}/\sqrt{n}\to 0$ (Fan and Li, 2001; Zou, 2006).

#### 2.2.3 Model selection

The adjusted coefficient of determination ($R_{\mathrm{adj}}^{2}$), the Akaike information criterion (AIC), the Schwarz Bayesian information criterion (SBC), and the average square error (ASE) are cohesion criteria used to compare LASSO and ALASSO (γ=0.5 and γ=1) results in the model selection. They are called goodness-of-fit measurements, and for a statistical model this shows inconsistency between the observed and expected values (Maydeu-Olivares and García-Forero, 2010).

7 $$R_{\mathrm{adj}}^{2}=1-\left(1-R^{2}\right)\frac{n-1}{n-p-1}$$

In Eq. (7), $R^{2}$ shows the coefficient of determination, p is the total number of explanatory variables in the model not including the constant, and n shows the sample size. AIC (Akaike, 1974) and SBC (Schwarz, 1978) are

AIC=-2ll+2p,8SBC=nln⁡(SSE/n)+pln⁡(n).

“ll” shows the log likelihood, and SSE is the sum of square error. The ASE is another cohesion criterion.

9 $$\mathrm{ASE}=\frac{\sum_{i=1}^{n}{\left(Y_{\mathrm{new}}-\left({\hat{\beta }}_{0}+\sum_{i=1}^{p-1}{\hat{\beta }}_{j}X_{\mathrm{new},j}\right)\right)}^{2}}{n},$$

where $Y_{\mathrm{new}}$ and $X_{\mathrm{new}}$ express new data that are unusable to estimate the coefficients of β. The model having minimum AIC, SBC, and ASE values is determined to be the best when selecting the model.

The statistical evaluations were performed by using MEANS, CORR, GLM, and GLMSELECT procedures in SAS (2014). The R program was used to create a figure showing the correlations. The GLM procedure was used to eliminate age effect before performing OLS, and then the Ridge, LASSO, and ALASSO methods were applied.

**Table 1:** Descriptive statistics regarding live weight and some body measurements for male and female goats.

	Male (n=35)			Female (n=97)	
Variable	Mean ± SE	CV		Mean ± SE	CV
LW	$79.298\pm 1.986^{*}$	14.82		58.405±0.927	15.62
FW	$15.650\pm 0.144^{*}$	5.44		13.165±0.108	8.02
EL	19.980±0.440	13.04		20.036±0.351	17.21
HL	18.430±0.276	8.850		17.915±0.122	6.70
CW	$22.150\pm 0.253^{*}$	6.76		20.567±0.175	8.39
RH	$84.900\pm 0.886^{*}$	6.17		77.217±0.389	4.96
WH	$89.150\pm 1.010^{*}$	6.70		79.619±0.390	4.82
BH	$82.800\pm 0.949^{*}$	6.78		75.258±0.406	5.35
CD	$39.950\pm 0.489^{*}$	7.24		34.041±0.186	5.40
CG	$101.000\pm 1.115^{*}$	6.53		89.211±0.492	5.43
BL	$82.150\pm 0.740^{*}$	5.33		74.923±0.401	5.27
Age	$3.25\pm 0.741^{*}$	28.01		4.16±1.129	30.06

${}^{*}$ Differences from the females are statistically significant (P<0.05). SE: standard error; CV: coefficient of variation; LW: live weight; FW: forehead width; EL: ear length; HL: head length; CW: chest width; RH: rump height; WH: withers height; BH: back height; CD: chest depth; CG: chest girth; BL: body length.

## 3 Results

There were 35 male (26.52 %) and 97 female (73.48 %) goats in the study. Descriptive statistics regarding LW and biometric measurements (CW, RH, WH, BH, CD, BL, FW, EL, HL, CG, and age) and the results of univariate analysis of variance for all of variables in both genders are given in Table 1. It was observed that there were significant differences (P<0.05) between the genders for all the biometric measurement of Hair goats, except for EL and HL.

**Figure 1:**
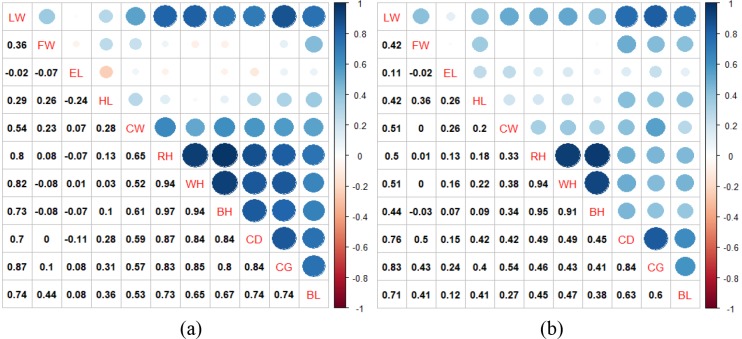
Pearson correlation coefficients between live weight and biometric body measurements for male **(a)** and female **(b)** goats.

The analyses were made after the data were corrected according to age. Pearson correlation coefficients displaying relationships between live weight and body measurements of Hair goats are presented by gender in Fig. 1. The values for males are shown in Fig. 1a, and those for females are shown in Fig. 1b. In Fig. 1, correlation coefficients greater than 0.5 were found to be statistically significant for males (P<0.01); whereas for females, coefficients greater than 0.26 were significant (P<0.01). There were correlation coefficients of over 0.8 between the explanatory variables in both genders, which made these data suitable for examination.

**Table 2:** OLS coefficients in multiple linear regression, tolerance, and VIF values for male and female goats.

	Male				Female		
Variable	Coefficients	TVs	VIF		Coefficients	TV	VIF
Intercept	$(-145.47\pm 31.923{)}^{b}$		0		$(-119.73\pm 11.65{)}^{b}$		0
FW	(2.972±2.634)	0.164	6.08		(0.437±0.565)	0.574	1.74
EL	(-0.546±0.482)	0.502	1.92		$(-0.311\pm 0.144{)}^{a}$	0.830	1.21
HL	(0.809±0.771)	0.523	1.91		(0.312±0.459)	0.673	1.49
CW	(0.648±0.962)	0.320	2.51		(0.637±0.341)	0.591	1.70
RH	(0.731±1.490)	0.013	79.67		(0.121±0.500)	0.056	17.90
WH	$(2.060\pm 0.696{)}^{a}$	0.050	20.93		(0.523±0.345)	0.094	10.69
BH	(-1.467±1.160)	0.020	51.36		(-0.383±0.370)	0.079	12.67
CD	(-1.880±1.054)	0.090	11.24		(0.104±0.514)	0.229	4.36
CG	$(0.875\pm 0.377{)}^{a}$	0.134	7.47		$(0.992\pm 0.198{)}^{a}$	0.221	4.52
BL	(0.272±0.549)	0.143	7.00		$(0.611\pm 0.162{)}^{a}$	0.500	1.20
RMSE	3.963				4.429		
$R^{2}$	0.946				0.789		
$R_{\mathrm{adj}}^{2}$	0.886				0.765		

${}^{a}$ p<0.05. ${}^{b}$ p<0.01. TVs: tolerance values; VIF: variance inflation factor values; LW: live weight; FW: forehead width; EL: ear length; HL: head length; CW: chest width; RH: rump height; WH: withers height; BH: back height; CD: chest depth; CG: chest girth; BL: body length; RMSE: root mean square error; $R^{2}$: the coefficient of determination; $R_{\mathrm{adj}}^{2}$: the adjusted coefficient of determination.

Regression coefficients, standard errors, tolerance values (TVs), and variance inflation factor (VIF) values are shown in Table 2 for both genders. The results revealed that all explanatory variables in the model explained 88.62 % of the variation in BL for males and 76.45 % for females. As shown in Table 2, there were VIF values of more than 10. VIF values for RH, WH, BH, and CD were found to be 77, 21, 51, and 11, respectively, in males. VIF values of RH, WH, and BH for females were 18, 11, and 13, respectively.

The coefficients and the standardized coefficients of Ridge, LASSO, and ALASSO (γ=0.5 and γ=1) in multiple linear regression are given in Table 3 for males and in Table 4 for females. The estimation equation for Ridge included all explanatory variables for both males and females, whereas LASSO and ALASSO (γ=0.5and γ=1) reduced the number of explanatory variables. In order to compare the methods some goodness-of-fit measurements such as $R_{\mathrm{adj}}^{2}$, AIC, SBC, and ASE are presented in Table 5, which shows that $R_{\mathrm{adj}}^{2}$ varied between 79.62 % and 90.48 % for males and between 74.95 % and 76.68 % for females.

**Table 3:** Coefficients and standardized coefficients of Ridge, LASSO, and ALASSO (γ=0.5 and γ=1) in multiple linear regression for male goats.

	Ridge			LASSO			ALASSO (γ=0.5)			ALASSO (γ=1)	
VN	Coefficients	Standardized		Coefficients	Standardized		Coefficients	Standardized		Coefficients	Standardized
		coefficients			coefficients			coefficients			coefficients
FW	3.767±1.373	0.273		4.570±2.285	0.330		4.556±0.88	0.330		4.564±0.984	0.331
EL	-0.108±0.438	-0.024		-0.204±0.158	-0.047		-2.135±0.106	-0.047		-0.241±0.131	-0.053
HL	0.462±0.739	0.0644		0.703±0.578	0.100		0.727±0.606	0.100		0.742±0.551	0.103
CW	-0.024±0.876	-0.003			–		–	–		–	–
RH	0.265±0.184	0.118			–		–	–		–	–
WH	0.796±0.209	0.796		1.623±0.557	0.826		1.802±0.353	0.917		1.928±0.331	0.981
BH	-0.007±0.203	-0.003		-0.362±0.192	-0.173		-0.527±0.369	-0.249		-0.637±2.408	-0.305
CD	-0.371±0.508	-0.091		-0.951±0.831	-0.234		-0.1.022±1.753	-0.252		-1.125±0.571	-0.277
CG	0.674±0.223	0.378		0.769±0.204	0.432		0.771±0.292	0.433		0.797±0.345	0.448
BL	0.096±0.325	0.036			–		–	–		–	–

VN: variable name; LW: live weight; FW: forehead width; EL: ear length; HL: head length; CW: chest width; RH: rump height; WH: withers height; BH: back height; CD: chest depth; CG: chest girth; BL: body length.

**Table 4:** Coefficients and standardized coefficients of Ridge, LASSO, and ALASSO (γ=0.5 and γ=1) in multiple linear regression for female goats.

	Ridge			LASSO			ALASSO (γ=0.5)			ALASSO (γ=1)	
VN	Coefficients	Standardized		Coefficients	Standardized		Coefficients	Standardized		Coefficients	Standardized
		coefficients			coefficients			coefficients			coefficients
FW	0.608±0.405	0.070		–	–		0.608±0.405	-0.056		–	-0.0720
EL	-0.184±0.116	-0.070		–	–		-0.184±0.116	–		–	–
HL	0.455±0.350	0.060		–	–		0.455±0.350	–		–	–
CW	0.728±0.245	0.138		0.276±0.077	0.052		0.728±0.245	0.064		0.276±0.077	0.074
RH	0.119±0.092	0.049		–	–		0.119±0.092			–	–
WH	0.197±0.106	0.083		0.127±0.056	0.054		0.197±0.106	0.085		0.127±0.056	0.092
BH	-0.034±0.096	-0.015		–	–		-0.034±0.096	–		–	–
CD	0.663±0.251	0.134		0299±0.59	0.060		0.663±0.251	–		0299±0.59	–
CG	0.641±0.094	0.340		0.936±0.119	0.497		0.641±0.094	0.579		0.936±0.119	0.580
BL	0.548±0.114	0.237		0.621±0.079	0.269		0.548±0.114	0.292		0.621±0.079	0.299

VN: variable name; LW: live weight; FW: forehead width; EL: ear length; HL: head length; CW: chest width; RH: rump height; WH: withers height; BH: back height; CD: chest depth; CG: chest girth; BL: body length.

**Figure 2:**
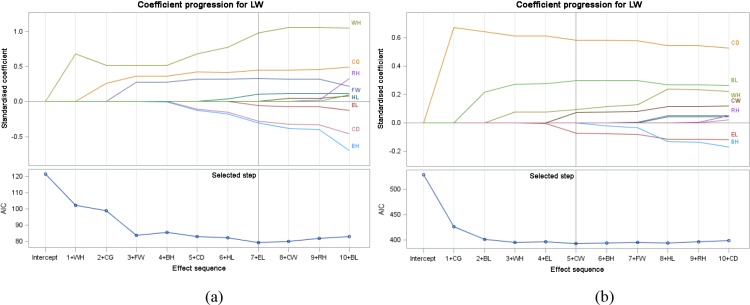
Coefficient progression with ALASSO (γ=1) for male **(a)** and female **(b)** goats.

**Table 5:** Goodness-of-fit criteria for estimation equations of Ridge, LASSO, and ALASSO (γ=0.5 and γ=1) for male and female goats.

	Male					Female			
GFC	Ridge	LASSO	ALASSO	ALASSO		Ridge	LASSO	ALASSO	ALASSO
			(γ=0.5)	(γ=1)				(γ=0.5)	(γ=1)
NV	10	7	7	7		10	5	5	5
$R^{2}$	0.893	0.935	0.938	0.940		0.7756	0.764	0.777	0.779
$R_{\mathrm{adj}}^{2}$	0.797	0.896	0.9028	0.905		0.7495	0.752	0.765	0.767
RMSE	5.590	3.783	3.682	3.625		4.5669	4.549	4.426	4.407
AIC	–	81.008	79.918	79.297		–	398.701	393.396	392.541
SBC	–	66.974	65.884	65.263		–	315.150	309.844	308.989
ASE	–	8.588	8.133	7.884		–	19.414	18.381	18.219

GFC: goodness-of-fit criteria; NV: number of variables; $R^{2}$: coefficient of determination; $R_{\mathrm{adj}}^{2}$: adjusted coefficient of determination; RMSE: root mean square error; AIC: Akaike information criterion; SBC: Schwarz Bayesian criterion; ASE: average square error.

In the current study we present the coefficient progression with AIC in Fig. 2a and b because we use AIC as a selection criterion. The selection process was done solely as visualized in Fig. 2. When the lowest AIC value was provided, the variable selection process was completed. As seen in Fig. 2, seven explanatory variables were selected for males: FW, EL, HL, WH, BH, CD, and CG. Five variables (FW, CW, WH, CG, and BL) were selected for females.

## 4 Discussion

The present results show that there was a significant difference between the genders in terms of body measurements in this study (P<0.05), with all measurements larger in males than females apart from ear length, despite females being on average older than the males. Similar results were reported by other researchers (Khan et al., 2014; Akbaş and Saatci, 2016). EL and HL were not measured in the study of Akbaş and Saatci (2016).

The correlation between LW and CG was found to be 0.87 for males and 0.83 for females (Fig. 1). The highest correlation coefficient with LW was revealed by CG for both genders. This was in agreement with the finding of previous studies (Pesmen and Yardimci, 2008; Cam et al., 2010; Tsegaye et al., 2013; Das and Yadav, 2015; Sam et al., 2016). The present study was focused the correlations between explanatory variables. Because there were high and significant correlations between explanatory variables, this study examined whether there was a multicollinearity problem. Previous studies have reported that when the tolerance values were less than 0.1 and VIF values were more than 10, the data had a multicollinearity problem (Montgomery et al., 2001; Yakubu, 2010; Dormann et al., 2013). According the results of OLS methods in MLR, the tolerance values found for RH, WH, BH ,and CD in males were 0.01255, 0.04779, 0.01947, and 0.08894, respectively, and corresponding VIF values were 77, 21, 51, and 11 (Table 2). Tolerance and VIF values for RH, WH, and BH in females were 0.05589, 0.09356, and 0.07891 and 18, 11, and 13 (Table 2). This result revealed that the current data set had a multicollinearity problem for both genders. It was emphasized by researchers that the multicollinearity implies that standard errors of regression coefficients are higher than expected, and, thus, it is difficult to find out the accuracy and robustness of the prediction models (Weisberg, 2005; Yakubu, 2009, 2010; Sangun et al., 2009).

In this study, where the variable selection for the data with multicollinearity is important, stepwise regression was not discussed because a previous study proposed that stepwise regression had some limitations and problems (Fan and Li, 2001; Shen and Ye, 2002; Whittingham et al., 2006). The body weight has been predicted from body structural and udder morphological traits in Frizarta dairy sheep, and it has been claimed that stepwise and LASSO regression selected the same variables with equal goodness-of-fit measurements (Kominakis et al., 2009). However, Kominakis et al. (2009) did not mention the multicollinearity problem.

In Ridge regression (in which coefficients of all explanatory variables are estimated), the adjusted $R^{2}$ values were 78.62 % for males and 74.94 % for females. Also, variable selection could not accomplished as reported in previous research (Pimentel et al., 2007; Topal et al., 2010; Ogutu et al., 2012; Orhan et al., 2016). Subsequently, LASSO and ALASSO for both γ=0.5 and γ=1 were performed to overcome the multicollinearity problem and also to select explanatory variables for the purpose of reducing model complexity. In all three methods, models consisted of seven variables for males and five variables for females. The adjusted coefficient of determination was 89.63 % for LASSO and 90.18 % and 90.48 % for ALASSO (for γ=0.5 and γ=1 methods, respectively) for male Hair goats (Table 3). ALASSO (γ=1) had the highest adjusted coefficient of determination. According to the model, FW, EL, HL, WH, BH, CD, and CG were selected as significant explanatory variables. The adjusted coefficient of determination of female Hair goats for the three methods was found to be 75.15 % (LASSO), 76.47 % (ALASSO, γ=0.5), and 76.66 % (ALASSO, γ=1) (Table 4). The method giving the highest adjusted $R^{2}$ was again ALASSO (γ=1), which selected the variables FW, CW, WH, CG, and BL. When all methods were evaluated in terms of an adjusted coefficient of determination, it was found that Ridge regression gave the lowest coefficient in both genders of Hair goats.

When considering goodness-of-fit measurements for all methods (RMSE, AIC, SBC, and ASE), except for Ridge regression, ALASSO (γ=1) had the smallest value in both male and female goats. From this finding it was concluded that the best model explaining the change in LW was ALASSO (γ=1) in both genders of Hair goats. This is the first study to examine the ALASSO method with multilevel linear regression method to predict live weight from some biometric measurements and to select variables. Consequently, this study revealed that the best method explaining the variation in LW of male and female Hair goats is ALASSO (γ=1). The fact that ALASSO was a better method than LASSO was consistent with the findings of previous researchers (Fan and Li, 2001; Zou, 2006; Huang et al., 2008; Ogutu et al., 2012). They proposed that the ALASSO method was more advantageous compare to LASSO method due to its oracle property.

In this study, the results from ALASSO (γ=1) revealed that WH had the highest significant effect on LW in male goats, and the second main significant effect was CG. These were in agreement with the findings of the previous study (Yakubu, 2009), whereas many studies propose CG as the most important predictor (Cam et al., 2010; Tsegaye et al., 2013; Sam et al., 2016; Das and Yadav, 2015). The analysis of data having a multicollinearity problem should be treated with caution since the problem has been shown to be associated with unstable estimates of regression coefficients (Montgomery et al., 2001; Yakubu, 2010; Dormann et al., 2013; Khan et al., 2014). This justifies the use of ALASSO methods for prediction. However, the results of female Hair goats showed that CG was the main significant effect in LW. The same result was supported by Kominakis et al. (2009), Cam et al. (2010), Tsegaye et al. (2013), and Das and Yadav (2015).

## 5 Conclusions

In this study, LW was predicted from biometric measurement with high accuracy for both male and female Hair goats by using ALASSO (γ=1). However, the variable selection was performed by ALASSO (γ=1), unlike in Ridge. New statistical techniques like penalized regression methods can be successfully implemented in the investigation of relationships between LW and biometric measurements in goat, sheep, cattle, fish, etc.

## Data Availability

Data sets are available upon request by contacting the correspondence author.
